# Fully covered self-expandable metal stent for intraprocedural or late-diagnosed Type-II endoscopic retrograde cholangiopancreatography-related perforations

**DOI:** 10.1186/s12876-022-02466-9

**Published:** 2022-08-14

**Authors:** Osman Bozbiyik, Bartu Cetin, Tufan Gumus, Fatih Tekin, Alper Uguz

**Affiliations:** 1 Department of General Surgery, Ege University School of Medicine, Genel Cerrahi Bornova, 35100 Izmir, Turkey; 2 Department of Gastroenterology, Ege University School of Medicine, Izmir, Turkey

**Keywords:** ERCP, Duodenal retroperitoneal perforation, Fully covered self-expandable metallic stent

## Abstract

**Background:**

Perforations related to endoscopic retrograde cholangiopancreatography (ERCP) are rare but life-threatening complications. The treatment of Type-II-periampullary perforations that develop during endoscopic sphincterotomy remains a topic of discussion. This study aimed to evaluate the usefulness of fully covered self-expanding metal stenting (FCSEMS) for treating Type-II perforations.

**Methods:**

The files of all patients who underwent the ERCP procedures between January 2015 and October 2021 were retrospectively reviewed; patients with Stapher Type-II perforation were included in the current study. Patients with FCSEMS were classified into two groups: those who underwent FCSEMS and those who were conventionally followed up. Moreover, patients with FCSEMS were classified into two subgroups: those who underwent simultaneous stenting and those who underwent late stenting. Mortality, surgical intervention, percutaneous drainage, length of hospital stay, and inflammatory markers were all compared between the groups.

**Results:**

Of the 9253 patients undergoing ERCP during the study period, 28 patients (0.3%) were found to have Type-II perforation. The mean age of these patients was 67.7 ± 3.9 years, and 15 patients were female. FCSEMS was performed on 19 patients, whereas 9 patients were on conventional follow-up. None of the patients developed mortality. In the conventional follow-up group, one patient required percutaneous drainage and one required surgical intervention. In contrast, none of the patients in the FCSEMS group required additional intervention. At a statistically significant level, the length of hospital stay was found to be shorter in the FCSEMS group. There was no difference in inflammatory markers between the two groups. In nine patients, FCSEMS was performed simultaneously, whereas, in ten patients, FCSEMS was performed later because they required a second intervention. These two subgroups did not differ in terms of outcomes.

**Conclusions:**

FCSEMS is a safe and effective treatment modality for patients with Type-II perforation. Moreover, it can be safely used in patients whose perforations are diagnosed during the ERCP procedure and in patients whose diagnoses are made after the procedure.

## Background

Endoscopic retrograde cholangiopancreatography (ERCP) is a breakthrough diagnostic and treatment modality for pancreatic and biliary diseases. Perforation is a rare but most serious life-threatening complication of the ERCP procedure occurring in 0.08%–1.5% of the patients undergoing ERCP [1–3]. There are four types of perforations according to Stapfer’s classification as follows: Type-I perforations are defined as injury to the duodenal lateral wall caused by endoscope maneuvers, Type-II as injury to the duodenal medial wall caused by sphincterotomy or precut, i.e., periampullary injury, Type-III as pancreatic or bile duct injuries, and Type-IV as the presence of air in the retroperitoneum. Surgery is frequently used to treat Type-I perforations, whereas conservative treatment is almost always successful in treating Type-III and Type-IV perforations [4]. However, there is no consensus in the literature regarding the treatment of Type-II perforations.

Sphincterotomy-induced Type-II perforations are the most common type of perforation. The management of Type-II perforations has changed over the years. Non-operative management of Type-II perforations is possible in 90% of cases if the patient selection is done carefully [5]. Endoscopic treatment methods, such as endoscopic clipping, plastic stenting, and nasobiliary stenting, have been defined to improve chances of success in the non-operative treatment of these perforations [6]. Fully covered self-expandable metal stents (FCSEMS) has been widely used in the non-operative management of sphincterotomy-induced perforations over the last decade. FCSEMS can close the perforated area, remove the bile, and be easily removed be after the perforation has healed [7].

In our clinic, we have been using FCSEMS to treat ERCP-induced Type-II perforations both during and after the procedure in our routine practice since 2015. In patients with Type-II perforation, intraprocedural FCSEMS appears to have potential advantages, according to a recent study [8]. However, a significant number of Type-II perforations can still be detected after the procedure. Despite some case reports demonstrating the feasibility of post-ERCP FCSEMS in patients with Type-II perforations, the volume of data on this subject is not adequate. Therefore, the present study intended to demonstrate the clinical efficacy of intraprocedural and late FCSEMS in patients with Type-II perforation.

## Methods

Upon obtaining the approval of the local ethics committee (Approval number: 20-7T/64), the electronic information system was reviewed to list all patients developing ERCP-induced perforation between January 2015 and October 2021. The perforations were then classified according to the Stapfer’s classification. Only patients with Stapfer Type-II perforation were included in the present study.

Demographic characteristics, procedural indications, systemic diseases, characteristics relating to previous surgical operations, bile duct anatomy and variations, diagnostic methods, diagnosis times, types of perforations, presence of stents, presence of underlying malignancies, post-procedural laboratory findings, radiological findings, lengths of hospital stay, interventional procedures performed, and presence of any performed surgery were among the patient data evaluated.

All ERCP procedures were conducted under CO2 insufflation. Routine antibiotic prophylaxis with 400 mg intravenous ciprofloxacin was administered to the patients in the pre-procedural period. ERCP-induced perforations can be detected during or after the procedure. Intraprocedural perforations can be detected in patients with luminal patency on endoscopy, opaque contrast agent leaking from the lumens on fluoroscopy, and presence of retroperitoneal or intraperitoneal gas shadows. In our standard treatment approach, if perforation is detected during the procedure, the procedure is stopped to identify the type of injury, and the appropriate treatment is provided based on the type of injury identified. In cases where Type-II injury was detected during the procedure, after ensuring that the patient was in a state favorable for stenting, 40 × 10 mm or 60 × 10 mm FCSEMSs (Wallflex, Boston Scientific, Natick, Massachusetts, USA) were inserted. The length of FCSEMS was selected based on the location of the cystic duct. 40 mm FCSEMS was used if the cystic duct was opened distal to the common bile duct. 60 mm FCSEMS was used if the patient had cholecystectomy or if the cystic duct was proximal to the common bile duct.'

Patients who had no perforation during the procedure but developed abdominal pain afterward were admitted to our clinic and closely monitored. Oral intake was stopped, intravenous (IV) hydration was started, and complete blood count and biochemical blood values were assessed in these patients. Abdominal computed tomography (CT) with oral opaque contrast agent was performed on patients whose abdominal pain did not improve. After the procedure, an approach was chosen based on the clinical findings of Type-II perforation patients. Patients with ERCP-induced perforation were treated by a multidisciplinary team of general surgeons, gastroenterologists, and radiologists in our routine practice. The decision to perform an intervention on patients with perforation diagnosed after the procedure was made on the basis of the clinical and radiological findings in each patient. Patients with mild abdominal findings on physical examination, no significant leukocytosis, or no systemic inflammatory response syndrome findings (patients with milder clinical findings) were conventionally followed up. However, patients with significant sensitivity or an opaque contrast agent leaking into the limited area of the retroperitoneal space on CT received rescue FCSEMS as the second intervention. Percutaneous drainage was performed in patients with localized retroperitoneal collection, whereas surgical intervention was performed in patients with extensive peritonitis, significant opaque contrast agent leakage, and free intraperitoneal perforation findings on CT (intraperitoneal gas; intraperitoneal oral contrast; intraperitoneal free fluid or food collections; and discontinuity of the intestinal wall).

In all patients for whom non-surgical treatment was planned, bowel rest, antibiotherapy, proton-pump inhibitor, and monitorization of vital signs were initiated. Monitorization of full blood count/biochemistry parameters and close follow-up examinations were performed. Depending on the clinical course of each case, the multidisciplinary team made decisions about whether to continue medical monitoring, repeat CT examinations, and perform percutaneous drainage or surgery. The resumption of oral feeding was decided according to the patient's peritoneal irritation findings and bowel activity (eg, bowel sounds, flatus, bowel movement). The patients were discharged after confirmation of no signs of inflammation in blood and physical examination, and good general performance status.

Patients with Type-II perforation were classified into two groups: those who received conventional treatment and those who underwent FCSEMS. The patients who underwent FCSEMS were classified into two subgroups: those who underwent simultaneous FCSEMS and those who underwent late FCSEMS as a second intervention, and their outcomes were compared. The surgical intervention requirement and mortality rate were the primary outcomes. The length of the hospital stay, the highest C-reactive protein (CRP) and leukocyte values in the 48-h period following the procedure, and the need for percutaneous drainage were all recognized as secondary outcomes.

Olympus Duodenoscopes (TJF 150, TJF 160, Tokyo, Japan) were used to perform ERCP. Moreover, fully covered self-expandable metal stents (Wallflex, Boston Scientific, Natick, Massachusetts, USA) were used.

The present study was conducted in accordance with the ethical standards of the Declaration of Helsinki. Statistical analyses were performed using IBM SPSS for Windows, v.25 (IBM Corp., Armonk, N.Y., USA). Continuous variables were presented as mean ± standard deviation and the data were compared using the t-test. Categorical variables were expressed as numbers and percentages and analyzed using the Chi-square test or the Fisher’s exact test. P value of ≤ 0.050 was considered statistically significant for all hypotheses.

## Results

During the 7-year study period, a total of 9253 ERCP procedures were performed in our hospital. ERCP-induced Type-II perforations were found in 28 patients (0.3%). The study excluded patients with Type-I, Type-III, and Type-IV perforations. Of the 28 patients, 15 were females, and 13 were males. The mean age of the patients was 67.7 ± 3.9 years. The demographic characteristics of the patients are shown in Table 1.

**Table 1:** Demographic and clinical characteristics of the conventional therapy and the FCSEMS groups

	Conventional (n = 9)	FCSEMS (n = 19)	P
Age (y)	68 (60–77)	70 (33–88)	0.522
Sex (Male/Female)	5/4	8/11	0.505
ERCP İndication- choledocolithiasis	7 (77.8%)	16 (84.2%)	0.454
Malignancy	2 (22.2%)	1 (5.3%)	0.175
Abnormal papilla anatomy	2 (22.2%)	3 (15.5%)	0.678
Atypical papilla position	2	1	
Near the diverticulum	0	2	
Previous ERCP history	2 (22.2%)	6 (31.6%)	0.484
Previous surgery history	5 (55.6%)	10 (52.6%)	0.885
Distal gastrectomy and Bilroth II reconstruction	2	0	
Cholecystectomy	3	10	
Precut sphincterotomy	1 (11.1%)	6 (31.6%)	0.243
Pancreatitis	2 (22.2%)	5 (26.3%)	0.815

ERCP, Endoscopic retrograde cholangiopancreatography, FCSEMS, Fully covered self-expandable metal stent, Abnormal papilla anatomy, periampullary diverticulum or atypical papilla position

Indications for ERCP were choledocholithiasis in 23 patients, Sphincter oddi dysfunction in 2, IgG4-related sclerosing disease in 2, and malignancy in 1. Overall, 20 patients were undergoing the ERCP procedure for the first time, whereas 8 patients had a history of undergoing the ERCP procedure. Of these, six patients had previously undergone sphincterotomy. Of these six patients, two had balloon dilatation and four had re-sphincterotomy. Eleven patients had a history of cholecystectomy, two patients had a history of distal gastrectomy, and two had a history of reconstructive operation. Additionally, two patients had atypical localization of the papilla of Vater and two patients had juxtapapillary diverticulum next to the papilla of Vater. Moreover, seven patients had undergone precut sphincterotomy. Post ERCP pancreatitis was observed in seven patients. All were mild acute pancreatitis, which is characterized by the absence of organ failure and local or systemic complications, according to the modified Atlanta criteria [9].

Perforation was detected during the procedure in 10 patients (35.7%); of these, simultaneous FCSEMS was performed on nine patients. As cannulation was impossible in one patient who developed perforation during the precut, simultaneous FCSEMS could not be used. In this patient, a catheter extending from the common bile duct to the duodenum was placed by percutaneous transhepatic cholangiography, and ERCP was repeated and FCSEMS was placed. Rescue FCSEMS was placed to a total of 10 patients, by repeating ERCP again within 7–48 h in this patient and nine other patients whose perforation was detected after the procedure. Another nine patients with perforation detected after the procedure were treated with the conventional non-operative treatment rather than FCSEMS.

While comparing the groups in terms of the primary outcome, no surgical or percutaneous intervention was required for any patient who were treated with either simultaneous or late FCSEMS. Among the patients in the conventional follow-up group, one patient underwent percutaneous drainage for retroperitoneal collection and another patient underwent surgical treatment. The mean length of stay in the hospital was 8.7 ± 6.7 (2–34) days. Furthermore, the mean time it took for the FCSEMSs to be removed was 29.9 ± 17.3 (6–62) days. On the 52nd and 54th days, endoscopy was performed to end FCSEMS in two patients, and the stents were seen to fall off.

When the two groups were compared, the length of hospital stay in the FCSEMS group was significantly shorter than that of the conventional follow-up group (median length of hospital stay: 10 vs. 7 days, p = 0.012). None of the patients who underwent simultaneous or late FCSEMS required percutaneous intervention or surgical intervention (Table 2). Percutaneous drainage was performed on one patient and surgical intervention (surgical repair, cholecystectomy, T-tube drainage) was performed on another patient on the fifth day in the conventional follow-up group. In terms of the highest CRP and leukocyte values during the first 48 h, there was no difference between the conventional treatment and FCSEMS groups. In terms of length of hospital stay and highest leukocyte and CRP values in the first 48 h, there was no statistically significant difference between the subgroups of simultaneous FCSEMS and late FCSEMS (Table 3).

**Table 2:** Comparison of the conventional therapy and the FCSEMS Groups

	Conventional therapy (n = 9)	FCSEMS (n = 19)	P
Leukocyte-/μL (median)*	14.250 (5.280–23.970)	12.700 (6.370–26.890)	0.507
CRP-mg/L (median)*	198.00 (2.00–306.00)	116.00 (1.90–341.00)	0.121
Length of stay- day (median, min–max)	10 (7–34)	7 (2–14)	**0.012**
Need of percutaneous drainage or surgery (n)	2	0	
Mortality (n)	0	0	

The statistically significant values are written in bold

^*^The highest values in the first 7 days, FCSEMS, Fully covered self-expandable metal stent

**Table 3:** Comparison of the simultaneous FCSEMS and late FCSEMS subgroups

	Simultaneous FCSEMS (n = 9)	Late FCSEMS (n = 10)	P
Age (y)	70 (33–88)	71 (36–83)	0.693
Sex (male/female)	4/5	4/6	0.605
Precut sphincterotomy	4 (44.4%)	2 (20.0%)	0.252
Pancreatitis	1	4	0.153
Leukocyte-/μL (median)*	9.800 (6.370–26.890)	15.605 (7.570–23.020)	0.369
CRP-mg/L (median)*	97.90 (1.90–239.00)	120.50 (3.33–341.00)	0.327
Length of stay-day (median, min–max)	2 (2–13)	7 (3–14)	0.133
Need of percutaneous drainage or surgery (n)	0	0	
Mortality (n)	0	0	

^*^The highest values in the first 7 days, FCSEMS, Fully covered self-expandable metal stent

## Discussion

According to the present  study, patients with Type-II ERCP-induced perforation can mostly be treated non-operatively and FCSEMS is an effective method for treating patients with Type-II ERCP-induced perforation. The results of this study showed that FCSEMS is not only effective in Type-II perforations detected during the procedure but also in perforations detected later after the procedure, and that FCSEMS significantly reduces the length of hospital stay.

To the best of our knowledge, this is the first study to compare late FCSEMS with simultaneous FCSEMS and conventional follow-up, and it found that both simultaneous and late FCSEMS can yield effective results. Case reports and a four-case series on the use of rescue FCSEMS have already been published [10, 11].

Recently, the treatment of ERCP-induced perforations has been going through significant changes. The treatment of ERCP-induced duodenal perforations is determined by the location and type of perforation as well as the patient's clinical condition. The type of perforation injury is one of the most important considerations in treatment. Stapfer et al. classified the perforations into four types according to the mechanism of injury and the anatomical position of the perforation. Being the most common type of perforation, Type-II perforations are periampullary perforations of the duodenal medial wall that are typically caused by biliary or pancreatic sphincterotomy or precut papillotomy [12]. Unlike Type-I perforations that mostly require surgical intervention, a non-operative approach is feasible in most patients with Type-II perforations [13]. Endoscopic treatment methods have also been defined, in addition to non-operative follow-up. Nasobiliary drainage, often known as stenting, is a procedure for draining biliary and pancreatic fluid to the duodenum [14]. FCSEMS can be used to cover the perforated line, thereby preventing leaking while still allowing for biliary drainage. Similar to the esophagus and sleeve gastrectomy leaks, the fully covered metal stent prevents leakage due to the radial force that it applies to the leakage area, thereby limiting inflammation and peritonitis. Furthermore, epithelialization through the stent can accelerate the healing process. As a result, we believe that FCSEMS should be used in both cases where perforations are discovered during the procedure and cases where perforations are discovered thereafter.

Odemis et al. compared 10 patients who received intraprocedural FCSEMS for Type-II perforations to another group of 10 patients who received nasobiliary drainage; they reported that the patients who received FCSEMS required fewer analgesics, had lower leukocyte values on the first day, and spent less time in the hospital [8]. Tringali et al. also reported that they successfully treated 16 patients by performing simultaneous FCSEMS [15]. FCSEMS is useful for treating Type-II perforations, as observed in these two studies, which are similar to the findings of the current study. Except for one patient, all the FCSEMS procedures were performed simultaneously in these studies. Only 10 (35.7%) of the 28 patients in our series were diagnosed during the procedure, and 9 of them received simultaneous FCSEMS. Following the second ERCP, 10 patients underwent FCSEMS. In any of the patients who underwent FCSEMS, no surgical intervention or percutaneous drainage was required. The length of hospital stay in the group of patients undergoing FCSEMS was found to be significantly shorter than that of the group of patients on conventional follow-up. These findings indicate that FCSEMS can be performed safely in patients with Type-II ERCP-induced perforation and that FCSEMS is associated with shorter length of hospital stay.

The prognostic importance of early diagnosis of perforations has been demonstrated in previous studies [4, 16]. Stapfer Type-I perforations are easier to recognize during or immediately after ERCP. However, Stapfer Type-II perforations may be more difficult to diagnose during ERCP. The symptoms of these perforations are less distinct [17]. In the literature, there are a variety of series on the time it takes to diagnose ERCP-induced perforations. In the series where FCSEMS is applied simultaneously, it is seen that the rate of perforations diagnosed during the procedure is quite high [8, 15]. However, in many series in the literature, the rate of simultaneous diagnosis is not that high. Although Theopistos et al. were able to diagnose the perforations within the first 12 h in their 24-case series that investigated only Type-II perforations, the simultaneous diagnosis was made only in 3 (12.5%) patients [13]. Similarly, in a 61-case series by Kumbhari et al., the researchers were able to make the diagnosis during the procedure in only 10% of the cases and reported that the mean time until diagnosis was 23.6 ± 12.8 h [4]. In our study, 35.7% of the patients received simultaneous diagnosis of their perforations (n: 10). Simultaneous FCSEMS were performed on nine of these patients. In one patient who developed perforation during the precut, simultaneous FCSEMS could not be performed because intraoperative cannulation was not feasible. A total of 10 patients underwent ERCP within 7–48 h, and subsequently, rescue FCSEMS was performed. No surgical intervention or percutaneous drainage was required in any of the patients undergoing early or late FCSEMS. There was no difference between the groups of simultaneous FCSEMS and late FCSEMS in terms of length of hospital stay or other findings. According to these findings, which demonstrate the safety of FCSEMS in treating late-diagnosed perforations, patients with simultaneously diagnosed perforations and those with late-diagnosed perforations can benefit from the effective endoscopic treatment methods. There is not enough data in the literature about the timing of a second ERCP. In one of the largest series, Cochrane et al. placed FCSEMS to 23 patients with a 100% success rate for post-ERCP bleeding [18]. There must be a golden time for a second ERCP, as the perforation will progressively increase inflammation in the periampullary region. A second ERCP is defined as a safe procedure 2–4 days after failure of the first biliary cannulation [19, 20]. Since type 2 perforations already have sphincterotomy, cannulation is not difficult in very early period. In this study, all ERCP interventions were performed 7–48 h after the first procedure. It might be dangerous to perform the subsequent ERCP after 48 h of the first ERCP because of severe inflamation.

In our series, which is based on a multidisciplinary examination of the patients with perforations that were diagnosed after the procedure, patients with more stable clinical findings were conventionally followed up and those with more distinct findings in the clinical evaluation or imaging-based examinations were provided rescue FCSEMS as a second intervention. However, none of these patients who underwent rescue FCSEMS required additional intervention. In the conventional follow-up group, percutaneous drainage was required in one patient and surgical treatment in another patient. These data show that diversion treatment with FCSEMS is useful in Type-II perforations. Similar to our series, in a 10-case series by Odemis et al., a 16-case series by Tringali et al., and a 15-case series by Trikudanatan et al., the success rates in FCSEMS of perforations were reported to be 100% in both preventing mortality and the need for surgical intervention [8, 15, 21]. In 17 of the 18 patients with Type-II perforation, Bill et al. used endoscopic treatment with FCSEMS or plastic stents, and found that percutaneous drainage was required in one patient who received a plastic stent, but no additional intervention was required in any of the patients who received FCSEMS [22]. To prove that FCSEMS can reduce the need for surgical intervention, further series with larger sample sizes are needed.

An optimum time for FCSEMS extraction has not been clearly established. It has been stated that it is a safe procedure to remove FCSEMS within 30 days [10]. In previous series where FCSEMS was applied for ERCP perforation, the removal of FCSEMS was reported after a median duration of 10 and 43 days [8, 15]. In our series, the mean time to stent removal was 29.9 days, and no complications related to this procedure were observed.

The most important limitations of the current study include its single-centered and retrospective nature as well as the small sample size that it investigates. Furthermore, FCSEMS was performed on all patients with perforation detected during the procedure and only to those whose clinical or laboratory findings were more distinct in the other group of patients with late-diagnosed perforation, resulting in non-homogeneity across the groups. However, due to ethical and clinical difficulties in performing the study, creating a prospective and multicenter study on a subject like post-ERCP perforation is extremely difficult. Despite these limitations, the current study is one of the largest series of cases in the literature and the first to investigate late FCSEMS.

## Conclusions

In clinically stable patients with Type-II ERCP perforations, non-operative treatment is usually possible. For patients with Type-II perforation, FCSEMS is a safe and efficient treatment option. FCSEMS is a safe treatment option for patients who have been identified with Type-II ERCP-induced perforation during the procedure as well as for those with late-diagnosed perforation.

## Data Availability

The dataset generated and analysed during the current study is available from the corresponding author upon reasonable request.

## Abbreviations

ERCP: Endoscopic retrograde cholangiopancreatography
FCSEMS: Fully covered self-expandable metal stent
